# Optic nerve regeneration in larval zebrafish exhibits spontaneous capacity for retinotopic but not tectum specific axon targeting

**DOI:** 10.1371/journal.pone.0218667

**Published:** 2019-06-20

**Authors:** Beth M. Harvey, Melissa Baxter, Michael Granato

**Affiliations:** Department of Cell and Developmental Biology, Perelman School of Medicine, University of Pennsylvania, Philadelphia, PA, United States of America; National Eye Centre, UNITED STATES

## Abstract

In contrast to mammals, retinal ganglion cells (RGC) axons of the optic nerve even in mature zebrafish exhibit a remarkable capacity for spontaneous regeneration. One constraint of using adult zebrafish is the limited ability to visualize the regeneration process in live animals. To dynamically visualize and trace the degree of target specific optic nerve regeneration, we took advantage of the optical transparency still preserved in post developmental larval zebrafish. We developed a rapid and robust assay to physically transect the larval optic nerve and find that by 96 hours post injury RGC axons have robustly regrown onto the optic tectum. We observe functional regeneration by 8 days post injury, and demonstrate that similar to adult zebrafish, optic nerve transection in larval zebrafish does not prominently induce cell death or proliferation of RGC neurons. Furthermore, we find that partial optic nerve transection results in axonal growth predominantly to the original, contralateral tectum, while complete transection results in innervation of both the correct contralateral and ‘incorrect’ ipsilateral tectum. Axonal tracing reveals that although regenerating axons innervate the ‘incorrect’ ipsilateral tectum, they successfully target their topographically appropriate synaptic areas. Combined, our results validate post developmental larval zebrafish as a powerful model for optic nerve regeneration, and reveal intricate mechanistic differences between axonal growth, midline guidance and synaptic targeting during zebrafish optic nerve regeneration.

## Introduction

Axons of retinal ganglion cells (RGCs) extend from the retina into the brain, and together with associated glia form the optic nerve which transmits complex visual information into the visual centers of the brain. In mammals, damage to the optic nerve via injury or disease is irreversible, due to the well-documented challenges that contribute to the poor capacity for spontaneous regeneration throughout the mammalian Central Nervous System (CNS) (reviewed in [1]). The major causes for the lack of spontaneous regeneration include the limited ability to mount an intrinsic response to sustain axonal growth, and the presence of a growth inhibitory environment [2–5]. For the optic nerve, this limited regenerative capacity is further compounded by injury induced cell death of RGCs, adding an additional layer of complexity to the regeneration process [6]. Over the past decade several neuron intrinsic and extrinsic signaling pathways have been identified that suppress cell death and boost axonal growth [7–12]. These findings documented robust axonal growth of injured RGC towards their central targets, and in very few instances partially functional responses [9,13,14]. However, the full complement of molecular mechanisms and pathways that guide regenerating RGC axons and control target synaptic selectivity is not well defined.

In contrast to the mammalian CNS, amphibians and teleost fish including goldfish and zebrafish exhibit a remarkable capacity for spontaneous regeneration following spinal cord and optic nerve injury [15–17]. In fact, using adult zebrafish to study CNS regeneration has revealed mechanisms mediating axonal growth, glial interactions and functional recovery after spinal cord lesion [18–22]. Moreover, optic nerve regeneration in adult zebrafish occurs independently of neurogenesis [23,24], providing a unique opportunity to study mechanisms that promote spontaneous regeneration independently of the confounds of neural survival and neurogenesis.

Additional advantages of the zebrafish system include the ease of genetic and pharmacological manipulations and the unique ability for live cell imaging due to the optical clarity. Since these qualities persist throughout development into the period of visual system functionality, retinotectal development and function has been studied in great detail in zebrafish [25,26]. It is therefore somewhat surprising that post developmental larval zebrafish, which offer the same technical advantages, have not been developed as a system to study the cellular and molecular underpinnings of spontaneous optic nerve regeneration. Here, we present a simple yet powerful assay to transect the optic nerve and monitor axonal and functional regeneration in larval zebrafish. We find that RGC axonal regeneration is rapid, with re-innervating axons entering the optic tectum by 96 hours post transection (hpt), independent of cell death or RGC proliferation. We find that following complete transection of the optic nerve, axons regrow to the correct contralateral as well as the ‘incorrect’ ipsilateral tectum. In contrast, partial optic nerve transection results in axonal growth predominantly to the original, contralateral tectum, suggesting that spared axonal fiber tracts are critical for regenerating axons to navigate towards their original targets. Furthermore, we demonstrate that growing RGC axons re-innervate their original topographic tectal area, and that by 8 days post transection (dpt) larvae display functional visual regeneration. Altogether, this robust optic nerve regeneration assay in the larval zebrafish provides a powerful approach to further probe the cellular mechanisms and molecular pathways that promote spontaneous CNS axonal regeneration.

## Methods

### Ethics statement

All experiments were conducted according to an Animal Protocol fully approved by the Uni- versity of Pennsylvania Institutional Animal Care and Use Committee (IACUC) on January 24, 2014, protocol number 803446. Veterinary care is under the supervision of the University Laboratory Animal Resources (ULAR) of the University of Pennsylvania.

### Zebrafish maintenance and transgenes

All transgenic lines were maintained in the Tübigen or Tupfel long fin genetic background and raised as previously described [27]. The *Tg(isl2b*:*GFP)* transgenic line was used visualize RGCs and their axons [28].

### Transection assay

To inhibit melanocyte pigmentation, larvae were raised in phenylthiourea (PTU, 0.2mM in E3 medium) in the dark at 29°C beginning at shield stage. At 5 days post fertilization, larvae were anesthetized in PTU E3 plus 0.0053% tricaine then mounted in 2.5% low-melt agarose (SeaPlaque, Lonza) prepared with PTU E3 plus 0.016% tricaine ventral-up on a glass microscopy slide. Optic nerve injuries were performed on an Olympus SZX16 fluorescent microscope using a sharpened tungsten needle (Fine Science Tools, Tip Diameter: 0.001mm, Rod Diameter: 0.125mm) to transect the region of the nerve distal to it exiting the eye, yet proximal to the optic chiasm. Following injuries, larvae were removed from the agarose, allowed to recover in Ringer’s solution with 0.2mM PTU for about 60 minutes, then returned to 0.2mM PTU, E3 medium at 29°C. Larvae were inspected for transection efficiency at 16–18 hpt, and except for when examining partial transections, only larvae with complete optic nerve transections with no visible intact axons remaining from the eye to the tectum were kept in to 0.2mM PTU, E3 medium at 29°C until fixation or live-imaging at later designated timepoints. To count the number of transected optic nerves with any axonal regrowth to the optic tecta, as well as any nerves exhibiting misguided axonal growth, larvae were anesthetized, mounted in 1.5% low-melt agarose and observed on a Zeiss Axio Imager M1 fluorescent compound microscope at 72 hpt. To quantify the extent of axonal regrowth to the optic tecta, larvae were anesthetized, mounted in 1.5% low-melt agarose and live imaged using confocal microscopy at 72 hpt and 96 hpt. The total intensity of GFP signal in the left and right tecta was quantified using Imaris software (Bitplane) and analyzed in Graphpad Prism for statistical analysis.

To perform partial optic nerve injuries, larvae at 5 days post fertilization were anesthetized and mounted in agarose as described above. Then, the left optic nerve was injured with varying severity, ranging from uninjured to completely transected. The right eye was enucleated. Larvae were removed from the agarose and allowed to recover as described above. To assess the extent of the optic nerve injury at 24 hpt, each larva was anesthetized, mounted in 1.5% agarose and live imaged using confocal microscopy then returned to 0.2mM PTU, E3 medium at 29°C. The intensity of GFP signal in the right tectum was quantified using Imaris software (Bitplane). Larvae with partial transections were then grouped into three categories. At 72 hpt, larvae were again anesthetized, mounted in 1.5% agarose and imaged using confocal microscopy.

### Immunostaining

Larvae were stained using methods modified from those previously described [29]. Briefly, larvae were fixed in 4% PFA in PBS overnight at 4°C. Larvae were washed in PBS + 0.25% Triton (PBT), incubated in 150mM Tris-HCl pH 9.0 for 15 min at 70°C, then washed in PBT. Larvae were permeabilized in 0.05% Trypsin-EDTA for 5 min on ice [30], washed in PBT, blocked in PBT containing 1% bovine serum albumin (BSA), 2% normal goat serum (NGS) and 1% dimethyl sulfoxide (DMSO), and then incubated in primary and secondary antibodies overnight at 4°C in PBT containing 1% BSA and 1% DMSO. Stained larvae were stored and mounted in Vectashield (Vector Laboratories) for imaging using confocal microscopy. Primary antibodies used were: mouse anti-GFP (JL-8, 1:200, BD Biosciences), rabbit anti-caspase-3 (1:500, BioRad), mouse anti-PCNA (pc10, 1:1000, Sigma), rabbit anti-phospho-histone H3 (1:500, Millipore). Secondary antibodies used were: goat anti-mouse Alexa 488 (1:500, Molecular Probes), goat anti-rabbit Alexa 594 (1:500, Molecular Probes), goat anti-mouse Alexa 594 (1:500, Molecular Probes).

### Lipophilic dye labeling of retinal projections

Larvae were fixed in 4% PFA in PBS overnight at 4°C. To angle the larvae so the desired retina was aligned with the injection needle, larvae were laid against a coverslip secured on a glass microscopy slide and kept moist with PBS. Retinas were pressure injected with either 0.5% DiI (Invitrogen) or DiD (Invitrogen) dissolved in DMF. Larger or smaller volumes were injected to fully fill the retina or label smaller regions of the retina, respectively. Larvae were kept overnight at room temperature in PBS to allow dyes to diffuse then mounted in 1.5% low-melt agarose to be analyzed and imaged using confocal microscopy.

### Confocal imaging, processing and analysis

Larvae were imaged on a Zeiss 880 confocal microscope using a 20× objective. Image stacks of optic chiasms or tecta were compressed into maximum intensity projections. Images for caspase-3, PCNA and PH3 stainings were of single transverse sections of retinas. RGCs co-staining with caspase-3, PCNA or PH3 were manually counted from individual optical sections and analyzed in Graphpad Prism for statistical analysis. All images were adjusted for brightness and contrast in Adobe Photoshop CS5, and color assigned using Fiji.

### Behavioral assay

Dark-flash induced O-bend responses of zebrafish larvae were elicited and recorded as described previously [31]. Larvae tested for O-bend responsiveness were placed in a 4×4 clear plexiglass grid of sixteen 0.9×0.9 cm wells filled with E3 medium mounted in a 6 cm petri lid. Larvae were illuminated obliquely from above with a white light LED bulb (PAR38 LED light; LEDlight.com) as well as from beneath by an array of infared LEDs (IR100 Illuminator removed from its housing; YYtrade). The entire experimental set up was isolated under a black cloak. To evaluate O-bend responsiveness, the white LED was extinguished, and images were recorded by a high-speed Motionpro camera (Redlake, Tucson, AZ, USA) at 1000·frames·s–1, for the initial 800 ms of each 1 s dark flash. Larvae were given 10 dark flashes, each 2 minutes apart. To test the overall health of the larvae, we also recorded their responsiveness to an acoustic stimulus (1100 Hz for 2 ms duration) after the dark flashes were administered. Larvae that did not respond to an acoustic stimulus were not included in O-bend analyses. Data were recorded into Graphpad Prism for statistical analysis.

## Results

### Optic nerve transection induces robust RGC axonal regeneration

To monitor the progression of optic nerve regeneration *in vivo*, we focused on 5 day old larvae as they are largely transparent yet possess retinotectal connectivity underlying robust visual functionality [32–34]. Already at 32 hours post fertilization RGC axons begin to exit the retina and project contralaterally across the midline at the optic chiasm [25,26]. While a small proportion of axons project to non-tectal arborization fields, more than 97% of RGC axons terminate in the optic tectum [35]. Within the optic tectum, RGC axons project topographically [25] and terminate into four tectal laminae [36] to ultimately reconstruct visual input from the retina to the tectum.

**Fig 1:**
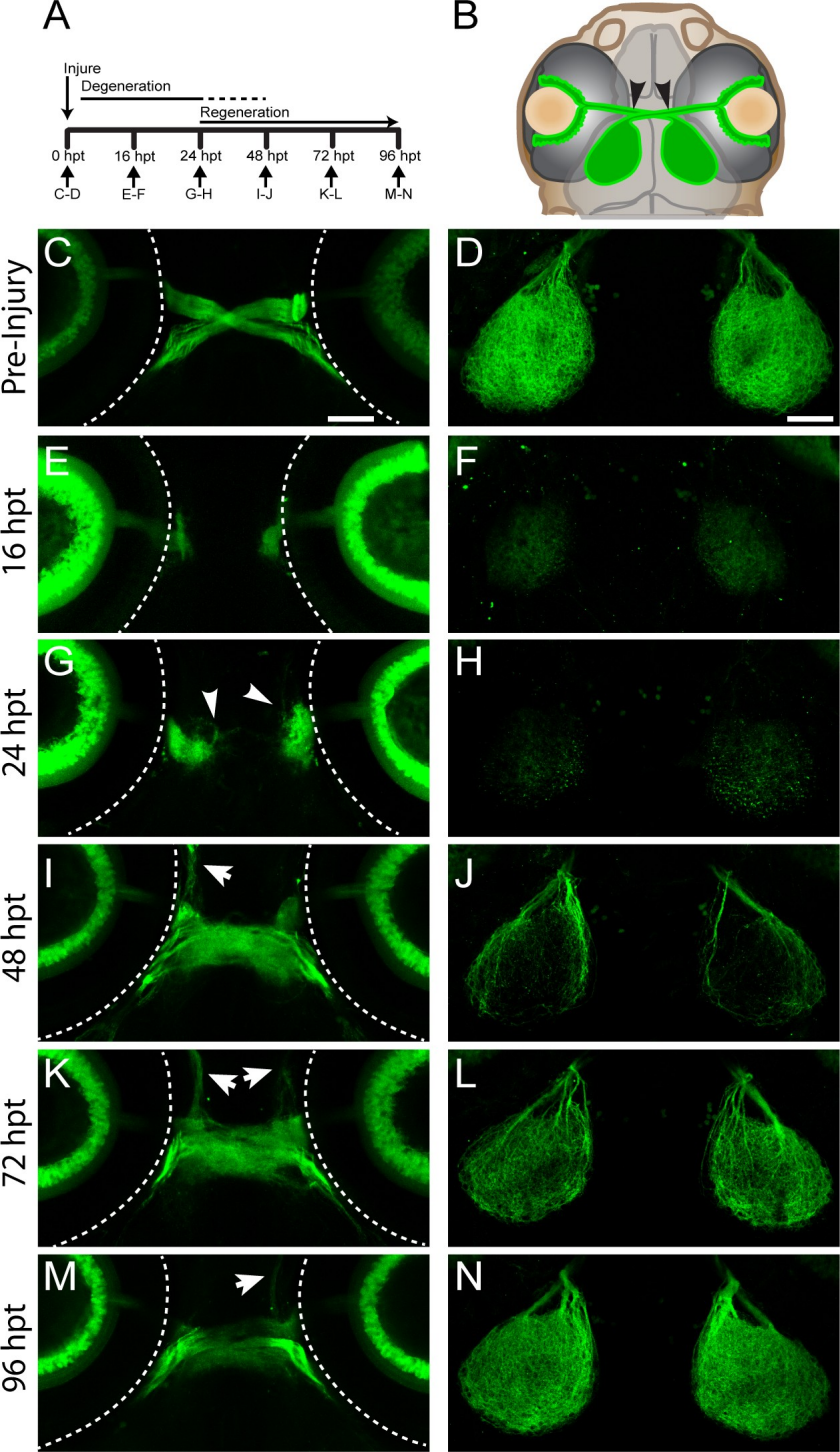
Regeneration of RGC axons in larval zebrafish. (A) Timeline of optic nerve regeneration. (B) Diagram of a *Tg(isl2b*:*GFP)* larva expressing GFP in RGCs and their axons. At 5dpf, optic nerves are transected with a sharpened tungsten needle proximal to the chiasm (black arrowheads). (C-D) Before injury, RGC axons cross the midline at the optic chiasm and innervate the contralateral tecta. Dashed lines indicate the outline of the eyes; scale bars = 50 μm. (E-F) Axonal growth from transected nerve at 16hpt is undetectable, while the portion of the nerve distal to the injury degenerates. (G-H) At 24 hpt, regrowing RGC axons begin to emerge from the proximal portion of the injured nerve (white arrowheads), while further degeneration continues. (I-J) By 48 hpt, regenerating axons project into the chiasm and re-innervate the tecta, though about 50% of transected nerves exhibit some misguided axonal growth (n = 81/160 nerves from 80 larvae, white arrows). (K-L) At 72 hpt, there is additional axonal growth to the tecta. (M-N) By 96 hpt, the axons within the chiasm fasciculate and robustly innervate the tecta (n = 160/160 nerves from 80 larvae). Representative images of chiasms and tecta for each timepoint are of the same fixed larva, though across timepoints are different larvae.

To characterize the process of optic nerve regeneration in larval zebrafish, we imaged optic nerves prior to transection and during the regeneration process using a transgenic line *Tg(isl2b*:*GFP)* expressing GFP in all RGC neurons and their axons (Fig 1A and 1B; [27]). Using a sharpened tungsten needle, we transected the optic nerve just proximal to the optic chiasm (Fig 1B, arrowheads). Prior to injury, RGC axons from one eye crossed the midline and exclusively innervated the contralateral optic tectum (Fig 1C and 1D). By 16 hpt, the portion of the nerve distal to the injury site was undergoing degeneration resulting in axonal fragments along the entire retinotectal trajectory and in the optic tectum (Fig 1E and 1F). At this time, we did not detect any axonal growth from the proximal nerve stump (Fig 1E). At 24 hpt, axonal degeneration had progressed further as evidenced by the presence of only axonal debris on the tectum (Fig 1H). Concomitantly, GFP-positive axons began to emerge from the proximal nerve stump (Fig 1G, arrowheads). By 48 hpt, regenerating axons crossed the optic chiasm at the CNS midline and began to re-innervate the tectum (Fig 1I and 1J). By 72 hpt, more regenerating axons entered the optic tectum and began to cover the entire tectum (Fig 1K and 1L). At 96 hpt, regenerating axons at the optic chiasm had a more fasciculated organization, and the optic tectum was more robustly re-innervated, likely reflecting the arrival of additional RGC axons (Fig 1M and 1N; S1A and S1B Fig). In about 50% of injured optic nerves, regenerating axons exhibited misguided growth directed away from optic chiasm (arrows, Fig 1I, 1K and 1M; n = 81/160 nerves from 80 larvae). Despite this presence of misguided RGC axons in about 50% of transected nerves (n = 81/160 nerves from 80 larvae), we observed predominant regrowth to the optic tecta in 100% of injured nerves (n = 160 nerves from 80 larvae), demonstrating that even after complete nerve transection, RGC axonal regrowth is extremely robust in larval zebrafish.

### Optic nerve regeneration occurs independently of RGC death or proliferation

**Fig 2:**
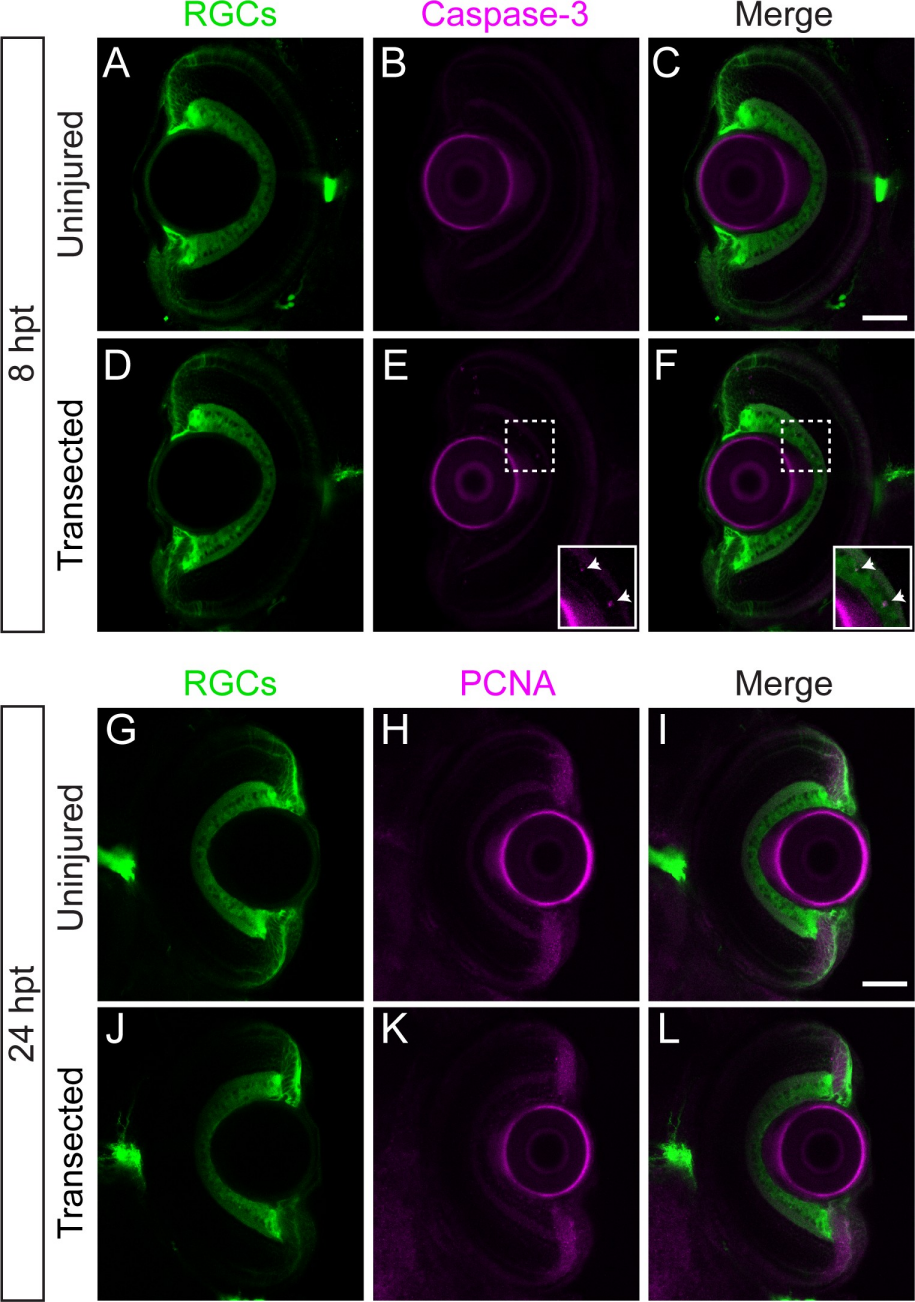
Optic nerve regeneration occurs in absence of significant RGC apoptosis or proliferation. (A-F) Transverse optical sections of retinas from *Tg(isl2b*:*GFP)* uninjured larvae (A-C; n = 8 retinas) or larvae with transected optic nerves (D-E; n = 8 retinas) at 8 hpt labeled with anti-active caspase-3 (magenta). Following optic nerve injury, very few RGCs co-label with caspase-3 (E-F inset; see Table 1). (G-L) Transverse sections of retinas from *Tg(isl2b*:*GFP)* uninjured larvae (G-I; n = 10 retinas from 5 larvae) or larvae with transected optic nerves (J-L; n = 14 retinas from 7 larvae) at 24 hpt labeled with anti-PCNA (magenta) show no apparent PCNA staining in the inner nuclear layer. Scale bars = 50 μm.

In larval zebrafish, both developmental and chemical injury induced RGC neurogenesis have been well documented [37–39], raising the possibility that optic nerve transection might lead to RGC cell death and neurogenesis. To evaluate these two processes, we used immunohistochemistry to assess RGC cell death (anti-active caspase-3) between 2 and 24 hpt (Fig 2A–2F; Table 1), and proliferation (anti-PCNA) at 24, 48, 72 and 96 hpt (Fig 2G–2L). Compared to retinas of uninjured larvae, following optic nerve transections did not reveal prominent RGC cell death (Fig 2A–2F; Table 1). Similarly, retinas of larvae with uninjured (24 hpt, n = 10 retinas from 5 larvae; 48 hpt, n = 10 retinas from 5 larvae; 72 hpt, n = 10 retinas from 5 larvae; 96 hpt, n = 10 retinas from 5 larvae) or with transected optic nerves (24 hpt, n = 14 retinas from 7 larvae; 48 hpt, n = 14 retinas from 7 larvae; 72 hpt, n = 14 retinas from 7 larvae; 96 hpt, n = 14 retinas from 7 larvae) did not show any apparent PCNA staining in the inner nuclear layer (Fig 2G–2L). Moreover, retinas of larvae with uninjured (48 hpt, n = 8 retinas from 4 larvae; 72 hpt, n = 12 retinas from 6 larvae; 96 hpt, n = 8 retinas from 4 larvae) or transected optic nerves (48 hpt, n = 10 retinas from 5 larvae; 72 hpt, n = 14 retinas from 7 larvae; 96 hpt, n = 8 retinas from 4 larvae) did not show any RGCs co-staining with PH3 (S2A–S2F Fig). Thus, similar to adult zebrafish yet unlike mammalian optic nerve injury, RGC regeneration occurs independently of RGC cell death and proliferation [24], thereby providing a valid model to study the process of axonal regeneration without the confounds of neurogenesis.

**Table 1:** Number of RGCs labeled with caspase-3. Mean number ± standard deviation of the mean of apoptotic cells. See Methods for more details on quantification.

Hours post transection (hpt)	Uninjured	Injured	Mann-Whitney test
**2**	**0 ± 0** (n = 8)	**0 ± 0** (n = 8)	**n.s.**
**4**	**0 ± 0** (n = 8)	**2.0 ± 1.6** (n = 8)	p ≤ 0.01
**8**	**0 ± 0** (n = 8)	**4.0 ± 2.3** (n = 8)	p ≤ 0.001
**24**	**0 ± 0** (n = 8)	**1.5 ± 2.3** (n = 8)	**n.s.**

### Functional recovery following optic nerve transection

**Fig 3:**
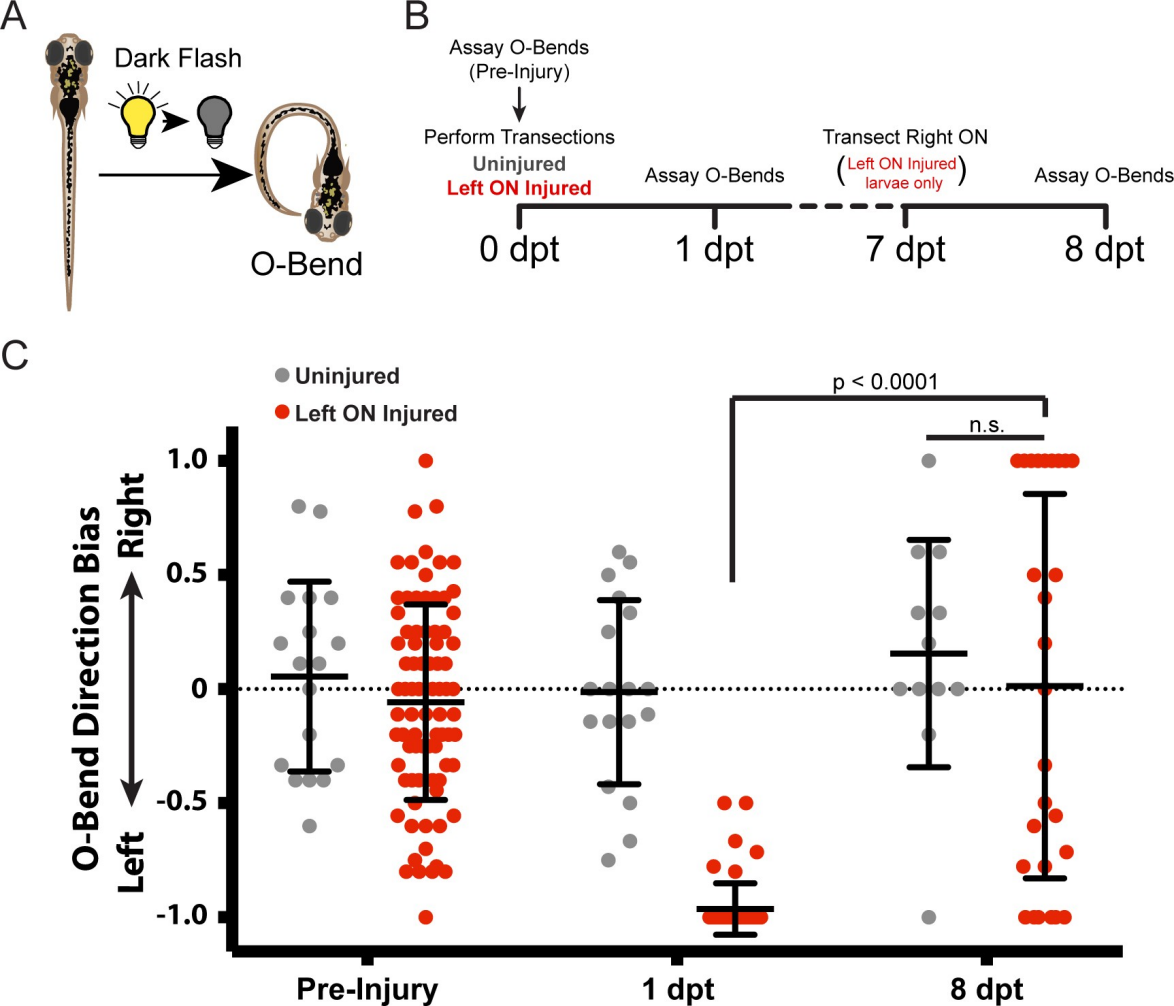
Functional regeneration of RGC axons. (A) Diagram illustrating the O-bend response elicited by suddenly extinguishing light (dark flash). (B) Timeline of events for testing functional regeneration. *Tg(isl2b*:*GFP)* larvae were uninjured or had the left optic nerve (ON) transected. O-bend responses to dark flashes were recorded prior to injuries at 0 dpt and then 1 dpt. At 7 dpt, the right ON was injured in larvae that originally received a left ON injury. A final assay for O-bend responses was performed at 8 dpt. (C) O-bend direction biases before (pre-injury; uninjured n = 18 larvae; left ON injured n = 83 larvae), after initial injuries were performed (1 dpt; uninjured n = 18 larvae; left ON injured n = 57 larvae), and after regeneration (8 dpt; uninjured n = 12 larvae; left ON injured n = 27 larvae). Before injuries, larvae perform O-bends with no directional bias. Larvae with a left ON injury at 1 dpt perform O-bends with a strong leftward directional bias, and by 8 dpt show no directional bias similar to uninjured larvae. ****p < 0.0001, Wilcoxon signed rank test; n.s., Mann-Whitney test.

**Fig 4:**
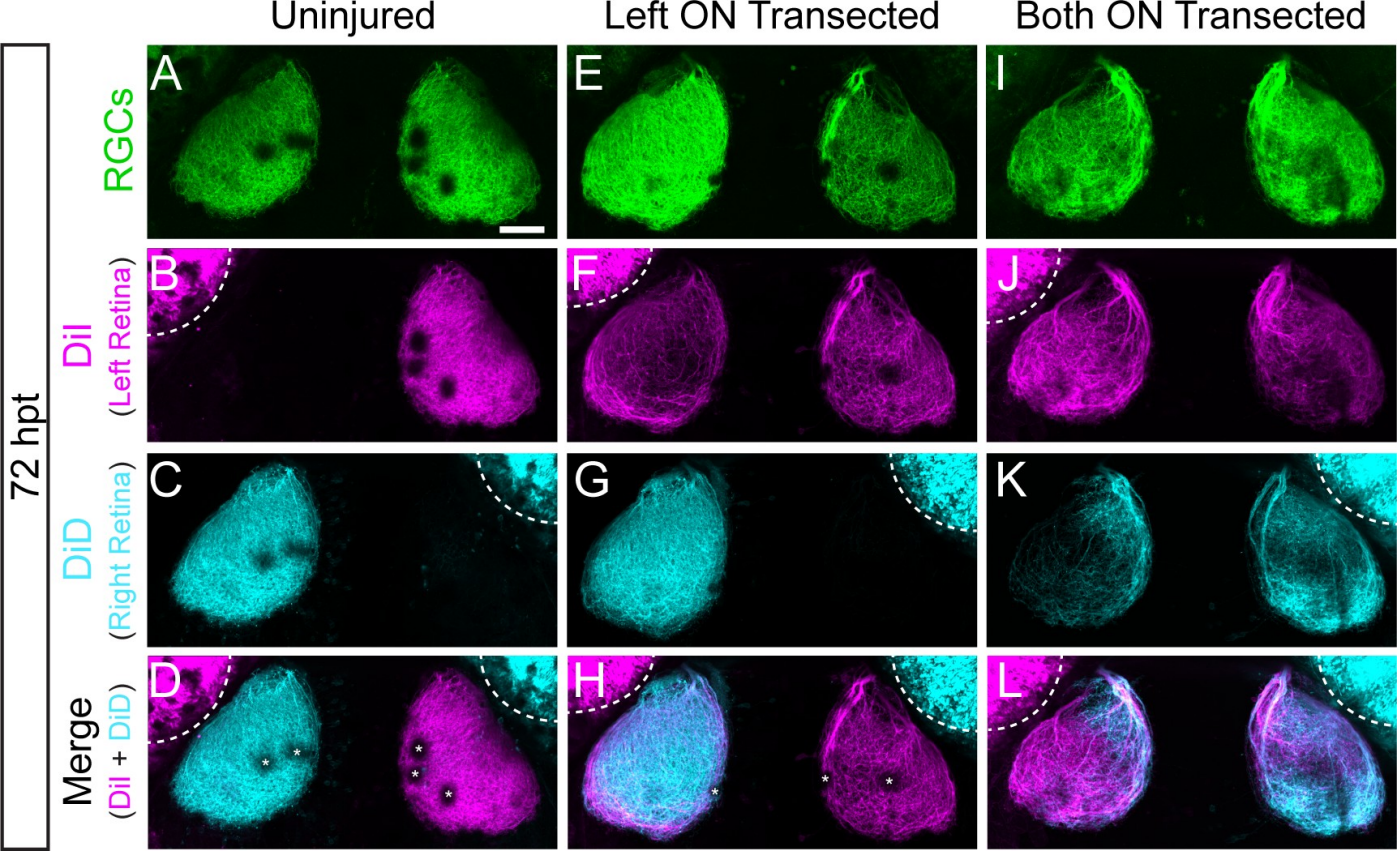
Transected RGC axons grow to ipsilateral and contralateral tecta. (A-L) Examples of RGC axonal tracings of Tg*(isl2b*:*GFP)* larvae at 72 hpt that were uninjured (A-D), received only a left optic nerve (ON) transection (E-H) or had both ON transected (I-L). Axons were traced by injecting the lipophilic dyes DiI and DiD into the left and right retina, respectively. RGC axons of uninjured nerves project to only contralateral tecta, while transected RGC axons grow to both ipsilateral and contralateral tecta. Asterisks indicate melanophores on the skin. Dashed lines outline dye fluorescence from the injected eye. Scale bar = 50 μm.

A hallmark and measurement of successful CNS regeneration is functional regeneration. Larval zebrafish perform several visually guided behaviors that have been studied extensively [32,34,40–43]. One such behavior is the O-bend behavior, which is robustly elicited in response to sudden darkness (dark flashes; Fig 3A) [31]. The O-bend is fully dependent on retinal function [44], and is quantifiable by several highly stereotypic kinematic parameters, including directional bias [31]. To determine if and to what extent larvae functionally recover from optic nerve transection, we subjected larvae to sudden dark flashes to elicit O-bends prior to optic nerve transection, one day then eight days post injury, and monitored the directional bias of the O-bends (Fig 3B and 3C). Importantly, transection of both left and right optic nerves dramatically reduced larval survival due to their inability to see and ingest food, precluding us to evaluate functional regeneration following bilateral optic nerve transection. We therefore transected only the left optic nerve, leaving the right optic nerve intact to allow larvae to successfully feed for the next six days. On the seventh day, i.e. one day prior to testing for functional regeneration at 8 dpt, we transected the right optic nerve of the experimental larvae, thereby allowing us to evaluate functional regeneration of only the originally transected left optic nerve (Fig 3B). In response to sudden darkness, uninjured controls performed left and rightwards O-bends without bias (Fig 3C, pre-injury; uninjured n = 18 larvae; left ON injured n = 83 larvae). One day after transection of the left optic nerve, when axonal degeneration occurs (Fig 1G and 1H), O-bend response bias was dramatically shifted leftward (Fig 3C, 1 dpt; uninjured n = 18 larvae; left ON injured n = 57 larvae). As detailed below, following complete optic nerve transection, regenerating RGC axons grew to both the ipsilateral and contralateral tectum, thereby providing visual inputs to both tecta (Fig 4). Therefore, we expected that functional regeneration of the left optic nerve at 8 dpt, in the absence of input from the now transected right optic nerve, would manifest as a directionally unbiased O-bend response. Indeed, experimental larvae performed O-bend turns without left or rightwards bias, indistinguishable from uninjured control larvae (Fig 3C, 8 dpt; uninjured n = 12 larvae; left ON injured n = 27 larvae). Thus, in response to optic nerve transection, we observe robust visual regeneration.

### Midline guidance during optic nerve regeneration is impaired

To further characterize the process of axonal regeneration following optic nerve transection, we performed axonal tracing at 72 hpt by injecting the lipophilic dyes DiI and DiD into the left and right retina, respectively. In zebrafish, RGC axons from the retina project exclusively to the contralateral tectum (Fig 4A–4D; n = 8 larvae) [25,26]. In contrast, following transection of the left optic nerve while sparing the right optic nerve, axons from the right retina projected contralaterally, while regenerating RGC axons from the left retina grew into both the ipsilateral and contralateral tectum (Fig 4E–4H; n = 9 larvae). Similarly, following transections of both optic nerves, regenerating RGC axons from the left retina and the right retina grew into both tecta (Fig 4I–4L; n = 14 larvae). Thus, regenerating RGC axons frequently fail to cross the midline and instead extend onto the ipsilateral tectum, consistent with the idea that following optic nerve injury the guidance mechanisms promoting midline crossing are inactive.

**Fig 5:**
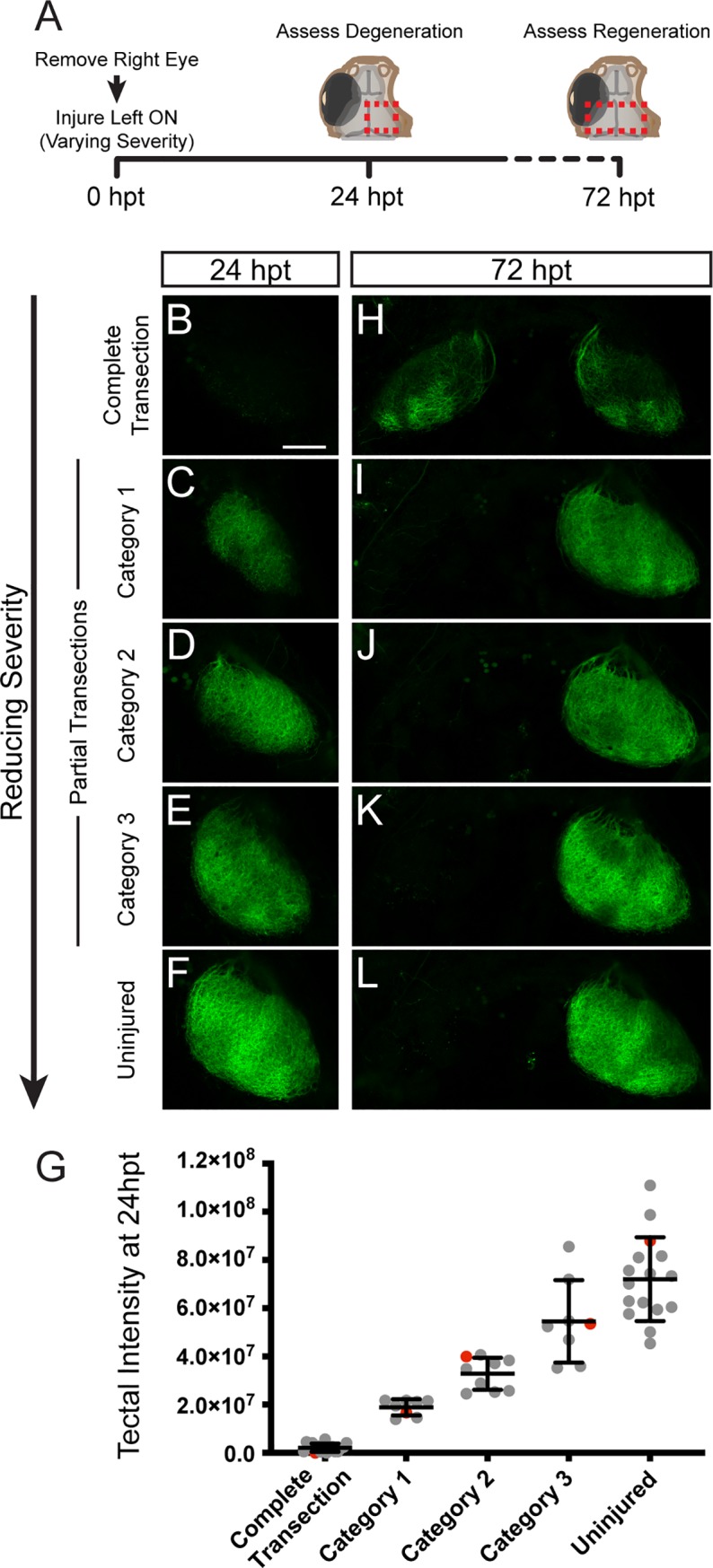
Partially injured RGC axons grow predominantly to contralateral tecta. (A) At 0 dpt, *Tg(isl2b*:*GFP)* larvae with the right eye removed received optic nerve transections ranging in severity from completely transected (n = 17 larvae), partially transected (Category 1, n = 8; Category 2, n = 9; Category 3, n = 8) to uninjured (n = 16). The extent of the transections was assessed at 24 hpt and regeneration to the tecta was examined at 72 hpt. Red dashed boxes indicate the imaged tectal areas. (B-F) Decreasing the severity of the transection spares more intact axons in the tectum at 24 hpt as shown by the GFP expression of RGC axons. (G) Quantification of the intensity of GFP signal in the right tectum at 24hpt. Red data points indicate the larvae represented in (B-F) images. (H-L) Reducing the severity of the injury reduces inappropriate growth to the ipsilateral tectum. Scale bar = 50 μm.

We then asked whether providing a scaffold of correctly projecting axons within the injured nerve would reduce or even prevent inappropriate ipsilateral growth. For this we removed the right eye and then performed partial transections of the left optic nerve, reducing the severity from completely transected to uninjured. At 24 hpt, we quantified the total RGC axonal GFP signal in the tectum to obtain a more quantitative measurement of the severity of partial optic nerve transections. We then grouped the partial transections based on their severity i.e. tectal GFP signal, and examined axonal regeneration to the tecta at 72 hpt (Fig 5A). We observed that reducing the severity of injury and hence increasing the amount of spared RGC axons (Fig 5B–5G), decreased the fraction of regenerating axons extending into the inappropriate ipsilateral tectum (Fig 5H–5L). Together, these data demonstrate that after complete transections, regenerating RGC axons fail to correctly navigate the CNS midline but that the presence of intact axonal tracts greatly facilitates regeneration to the original contralateral tectum.

### Regenerating RGC axons maintain target specificity in ipsilateral and contralateral tecta

**Fig 6:**
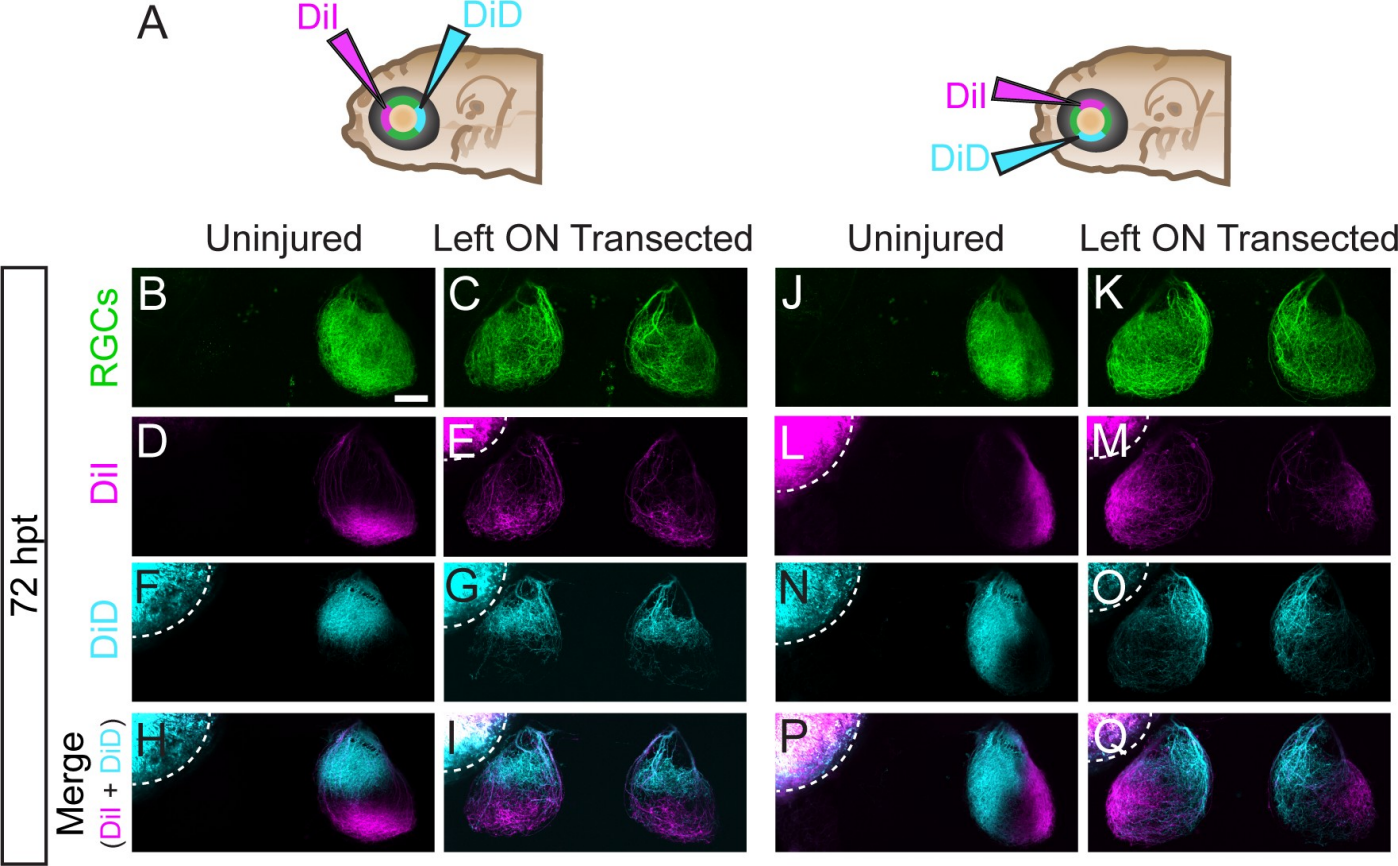
Regenerating RGC axons terminate in correct topographic regions. (A) At 72 hpt, small populations of RGCs were labeled by DiI and DiD injections into anterior and posterior, or dorsal and ventral quadrants of the left retinas of *Tg(isl2b*:*GFP)* that were uninjured or received left optic nerve (ON) transection. The right eye was removed to facilitate analysis. (B-Q) Following optic nerve transection, RGC axons project to both the ‘incorrect’ ipsilateral and contralateral tecta yet maintain the same correct topographic specificity as uninjured RGCs; (B-I) anterior RGCs to posterior tectum and posterior RGCs to anterior tectum (uninjured, n = 22 nerves from 22 larvae; left ON transected, n = 21 nerves from 21 larvae), as well as dorsal RGCs to ventral tectum and ventral RGCs to dorsal tectum (uninjured, n = 23 nerves from 23 larvae; left ON transected, n = 16 nerves from 16 larvae). Dashed lines outline fluorescence from the injected eye. Scale bar = 50 μm.

During development, RGC axons terminate in a topographically stereotyped order on the tectum, such that axons originating from cell bodies located in the anterior-ventral retina project to the posterior-dorsal tectum, and conversely axons originating from cell bodies located in the posterior-dorsal retina projecting to the anterior-ventral tectum [25]. To examine whether regenerating axons retain the capacity to return to their original topographic targets, we labeled small populations of RGCs in either the anterior-posterior or dorsal-ventral quadrants of the left retina with DiI and DiD, respectively (Fig 6A). To unequivocally determine the tectal targeting of regenerating left optic nerve axons, we removed the right eye. We found that identical to uninjured controls, regenerating RGC axons projected to expected topographic targets, with RGCs in the anterior quadrant of the retina projecting to the posterior tectum and RGCs in the posterior quadrant projecting to the anterior tectum (Fig 6B–6I; uninjured, n = 22 nerves from 22 larvae; left ON transected, n = 21 nerves from 21 larvae). Similarly, dorsal RGC axons projected to the ventral tectum, while ventral RGC axons projected to the dorsal tectum (Fig 6J–6Q; uninjured, n = 23 nerves from 23 larvae; left ON transected, n = 16 nerves from 16 larvae). Moreover, we found that regenerating axons projected to their topographically correct targets irrespectively of whether they grew to the correct contralateral tectum or to the incorrect ipsilateral tectum (Fig 6H, 6I, 6P and 6Q). Thus, we observe that once regenerating RGC axons reach the tectum, they re-establish topographic target specificity. Altogether, these data demonstrate that for regenerating optic nerve axons in zebrafish, growth across the CNS midline, but not topographic targeting, represents a significant challenge.

## Discussion

For decades, adult teleost fish have been utilized as a model organism for studying optic nerve regeneration due to their remarkable regenerative capacity [16,17,45,46]. Conversely, teleost embryos have been used extensively to study embryonic development and perform genetic screens, which when combined with its optical clarity enabling unparalleled live imaging capabilities altogether have made zebrafish embryos a prime model to study the molecular genetic mechanisms underlying the development of the retinotectal system [47–50]. Here we combine the advantages of post developmental larvae that possess a functional visual system yet have retained optical transparency with a robust transection assay to rapidly measure axonal and functional optic nerve regeneration. We observe that RGC axonal regeneration in the larval zebrafish is rapid and occurs independently of cell death or RGC proliferation. We also find that following partial transections of the optic nerve, RGC axons grow predominantly to the contralateral tecta, while complete transections result in axons projecting to both the ipsilateral and contralateral tecta. In addition, we show that RGC axons re-innervate their appropriate topographic target areas regardless of whether they innervate the original contralateral tectum or the ‘incorrect’ ipsilateral tectum.

### Larval zebrafish recapitulate key hallmarks of adult optic nerve regeneration on an accelerated timeline

The time course of optic nerve regeneration in adult teleost fish has been described in great detail. Regenerating RGC axons begin sprouting around 72 hpt and first appear on the tectum about one week following injury, leading to functional regeneration approximately 3 weeks post injury [46,51,52]. We find that in larval zebrafish the entire regeneration process is on an accelerated timeline, with regenerating axons innervating the entire tectum by 72 hpt (Fig 1K and 1L), leading to robust visual regeneration already at 8 dpt (Fig 3C). In rodents, more than 80% of RGCs die within two weeks following injury and very few surviving RGCs extend axons farther than the injury site [6,7,53]. Importantly, like in adult zebrafish, optic nerve regeneration in larvae occurs independently of RGC cell death or proliferation (Fig 2) [24]. This, combined with its optical transparency and the ease of small molecule absorption makes larval zebrafish a powerful and time accelerated platform to decipher the mechanisms that promote spontaneous regeneration independently of the confounds of neural survival and neurogenesis.

### The mechanisms underlying topographic mapping but not midline crossing are active during optic nerve regeneration

Adult zebrafish have retained a significant capacity for optic nerve regeneration [23,52,54], however, if and to what extent these RGC axons return to their original, retinotopic position has not been examined. At the time we transect the optic nerve, RGC axons have not only established a functional retinotopic map, but have also terminated at one of four sublaminae located at different depths of the tectum [36]. While we did not examine whether regenerating RGC axons return to their original sublamina, we used DiI/DiD tracing to determine if they terminated on the tectum according to their original retinotopic position. We find that regenerating RGC axons faithfully re-innervate the appropriate dorso-ventral and anterior-posterior map position on the optic tectum (Fig 6). Furthermore, even when regenerating RGC axons project to the incorrect ipsilateral, they target their appropriate tectal positions, suggesting that the mechanisms underlying the initial retinotopic targeting are active during the process of regeneration. Teleost fish throughout their lifespan continue to add and replace RGC neurons along the retinal periphery [55,56] and the tectum grows only at the posterior border [15,56,57]. Although, we cannot fully exclude the contribution of axonal growth from any newly generated RGCs in the retinal periphery, our findings support the idea that mechanisms establishing proper retinotopic mapping are retained throughout the animal lifespan.

While we demonstrate that regenerating RGC axons have retained the ability to terminate according to the original retinotopic map, we also show that RGC axons fail to properly navigate the CNS midline. Rather than exclusively crossing the midline and innervating their original target, the contralateral tectum, regenerating axons frequently project to the ipsilateral tectum (Fig 4), suggesting that the molecular mechanisms that regulate proper midline crossing are absent or inactive. This is consistent with previous work in adult zebrafish and goldfish that revealed an increase of ipsilaterally projecting optic nerve fibers after crush injury [23,58].

In the late 1990s, genetic screens identified over 30 developmental mutants that fell into distinct groups based on the aberrant trajectories and pathfinding errors of their RGC axons [47,48]. Interestingly, ten of those mutants that make midline crossing errors display ipsilateral projection phenotypes strikingly similar to what we observe after optic nerve transection (Fig 4; reviewed in [49]). These mutants have since been cloned, and it is noteworthy that six of these mutants are caused by mutations in various components of the Shh signaling pathway, including *shh* [59], the *shh* receptor *smoothened* [60], the *shh* regulators *dzip1* [61] and *disp1* [62], and the downstream effectors *gli1* [63] and *gli2* [64]. Therefore, it is conceivable that *Shh* signaling might also regulate correct midline crossing during optic nerve regeneration, and it will be exciting to explore whether these genes indeed play functional roles during this process. Finally, despite the progress on promoting optic nerve regeneration in mammalian systems via *Pten/Socs3* deletions, proper guidance at the optic chiasm is still poor [8,13,65], suggesting a role for guidance systems at the midline. Thus, identifying the molecular pathways that control midline guidance during zebrafish optic nerve regeneration might also inform this process in mammals.

## Supporting information

S1 Fig RGC axonal growth during optic nerve regeneration. (A) The total intensity of the optic tecta of larvae that received optic nerve transections increases from 72 hpt to 96 hpt. Each data point represents a single larva and all error bars indicate ±SEM. (B) Lines connect the points (from A) representing the total intensity of the optic tecta for each larva with optic nerve transections at 72 and 96hpt. ****p < 0.0001, Student’s t-test of mean total tectal intensity.
(PDF)

S2 Fig RGC axonal growth during optic nerve regeneration. (A-F) Transverse sections of retinas from *Tg(isl2b*:*GFP)* uninjured larvae (A-C; n = 8 retinas) or larvae with transected optic nerves (D-F; n = 10 retinas) at 48 hpt labeled with anti-phosphorylated Histone H3 (magenta) show no RGCs co-staining with PH3. Scale bars = 50 μm.
(PDF)
