# Supplementary material for: Optic nerve regeneration in larval zebrafish exhibits spontaneous capacity for retinotopic but not tectum specific axon targeting
Source: PLoS One. 2019 Jun 20;14(6):e0218667. doi: 10.1371/journal.pone.0218667 (PMC6586344; doi:10.1371/journal.pone.0218667)
Supplement: S1 Fig — (A) The total intensity of the optic tecta of larvae that received optic nerve transections increases from 72 hpt to 96 hpt. Each data point represents a single larva and all error bars indicate ±SEM. (B) Lines connect the points (from A) representing the total intensity of the optic tecta for each larva with optic nerve transections at 72 and 96hpt. ****p < 0.0001, Student’s t-test of mean total tectal intensity. (PDF) [file pone.0218667.s001.pdf]

Figure S1. RGC axonal growth during optic nerve regeneration.

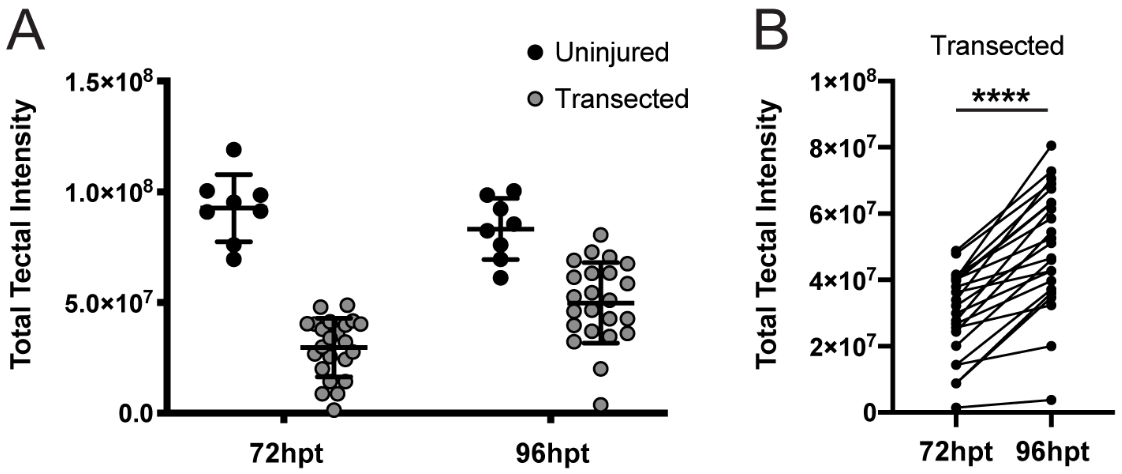

(A) The total intensity of the optic tecta of larvae that received optic nerve transections increases from 72 hpt to 96 hpt. Each data point represents a single larva and all error bars indicate ±SEM. (B) Lines connect the points (from A) representing the total intensity of the optic tecta for each larva with optic nerve transections at 72 and 96hpt. ****$p < 0.0001$, Student's t-test of mean total tectal intensity.
