# Supplementary material for: Optic nerve regeneration in larval zebrafish exhibits spontaneous capacity for retinotopic but not tectum specific axon targeting
Source: PLoS One. 2019 Jun 20;14(6):e0218667. doi: 10.1371/journal.pone.0218667 (PMC6586344; doi:10.1371/journal.pone.0218667)
Supplement: S2 Fig — (A-F) Transverse sections of retinas from Tg(isl2b:GFP) uninjured larvae (A-C; n = 8 retinas) or larvae with transected optic nerves (D-F; n = 10 retinas) at 48 hpt labeled with anti-phosphorylated Histone H3 (magenta) show no RGCs co-staining with PH3. Scale bars = 50 μm. (PDF) [file pone.0218667.s002.pdf]

Figure S2. RGC axonal growth during optic nerve regeneration.

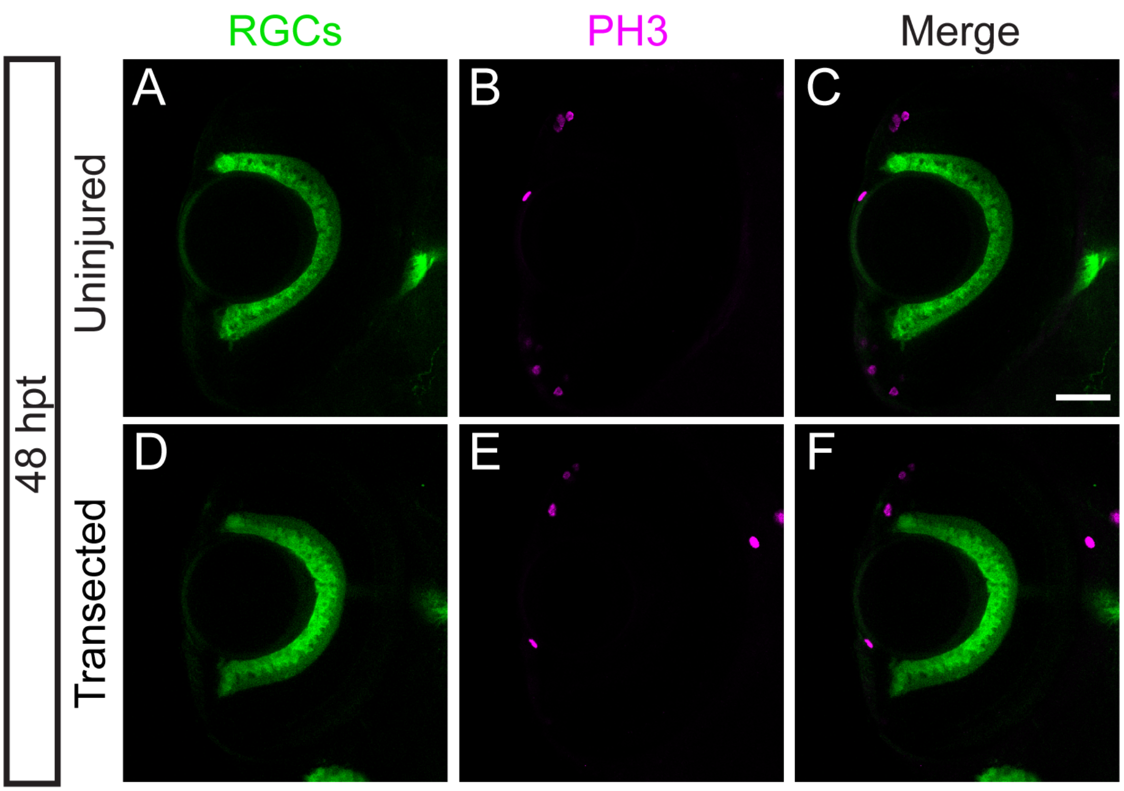

(A-F) Transverse sections of retinas from *Tg(isl2b:GFP)* uninjured larvae (A-C; n = 8 retinas) or larvae with transected optic nerves (D-F; n = 10 retinas) at 48 hpt labeled with anti-phosphorylated Histone H3 (magenta) show no RGCs co-staining with PH3. Scale bars = 50 μm.
